# Sexual Life in the Postpartum Period of Tunisian Women Following Episiotomy

**DOI:** 10.1192/j.eurpsy.2025.2305

**Published:** 2025-08-26

**Authors:** A. Hadj Salah, Y. Kilani, M. Ben Mbarek, A. Haouala, I. Batbout, F. Zaafrane, A. Mhalla, B. Amamou

**Affiliations:** 1 psychiatry, Fattouma Bourguiba Hospital, Monastir; 2 gynecology, Farhat Hached Hospital, sousse, Tunisia

## Abstract

**Introduction:**

The postpartum period can significantly affect sexual life, particularly for women who have undergone an episiotomy. This study examines the impact of episiotomy on sexual activity during the postpartum period.

**Objectives:**

To evaluate the sexual life of Tunisian women following episiotomy in the postpartum period.

**Methods:**

We conducted a prospective cohort study at the Gynecology and Obstetrics Department of Farhat Hached University Hospital in Sousse. The study included women who delivered vaginally with an episiotomy in 2020. Data were collected from medical records and delivery reports. Nine months postpartum, participants were contacted by phone to complete a questionnaire assessing their experiences with episiotomy and sexual satisfaction.

**Results:**

The final sample comprised 66 women with a mean age of 27 ± 2.8 years (range: 20-34 years).

Regarding sexual function, 84% of women were very or moderately satisfied with their sexual life before pregnancy, and 74% were satisfied during pregnancy. Most women resumed sexual activity between 7 and 8 weeks postpartum (78%), with 22% resuming before 8 weeks. Compared to pre-birth satisfaction, 54% of women reported no change in sexual satisfaction after childbirth, while 29% reported a decrease and 17% an increase.

Sexual satisfaction was statistically related to the mode of delivery, with 55% of women who had forceps delivery being dissatisfied or equally satisfied as dissatisfied (p=0.01).

Factors affecting postpartum sexual activity included fear of pain (32%), fear of another pregnancy (13%), perceived loss of body desirability (37%), body changes (31%), and excessive fatigue (24%).

**Conclusions:**

This study demonstrates that episiotomy can have a notable impact on postpartum sexual life. Several factors, including the type of suture used during delivery and the mode of delivery, influence women’s sexual satisfaction. These findings underline the importance of considering both physical and emotional aspects of postpartum recovery to improve the overall sexual health and well-being of women after childbirth.

**Disclosure of Interest:**

None Declared

